# Supplementary material for: The individuality of shape asymmetries of the human cerebral cortex
Source: eLife. 2022 Oct 5;11:e75056. doi: 10.7554/eLife.75056 (PMC9668337; doi:10.7554/eLife.75056)
Supplement: Supplementary file 1. [file elife-75056-supp1.docx]

| Eigen-group Index | 0 | 1 | 2 | 3 | 4 |
| --- | --- | --- | --- | --- | --- |
| Eigenvalues | 1^st^ | 2^nd^*–*4^th^ | 5^th^*–*9^th^ | 10^th^*–*16^th^ | 17^th^*–*25^th^ |
| Wavelength (mm) | N/A | 297.673 | 171.862 | 121.525 | 94.133 |
| Eigen-group Index | 5 | 6 | 7 | 8 | 9 |
| Eigenvalues | 26^th^*–*36^th^ | 37^th^*–*49^th^ | 50^th^*–*64^th^ | 65^th^*–*81^st^ | 82^nd^*–*100^th^ |
| Wavelength (mm) | 76.859 | 64.958 | 56.255 | 49.612 | 44.374 |
| Eigen-group Index | 10 | 11 | 12 | 13 | 14 |
| Eigenvalues | 101^st^*–*121^st^ | 122^nd^*–*144^th^ | 145^th^*–*169^th^ | 170^th^*–*196^th^ | 197^th^*–*225^th^ |
| Wavelength (mm) | 40.138 | 36.641 | 33.705 | 31.205 | 29.050 |
