# Supplementary material for: The individuality of shape asymmetries of the human cerebral cortex
Source: eLife. 2022 Oct 5;11:e75056. doi: 10.7554/eLife.75056 (PMC9668337; doi:10.7554/eLife.75056)
Supplement: Transparent reporting form [file elife-75056-transrepform1.pdf]

Westbrook Centre
Milton Road
Cambridge CB4 1YG, UK

P 01223 855340
W elifesciences.org
T @elife

eLife's transparent reporting form

We encourage authors to provide detailed information *within their submission* to facilitate the interpretation and replication of experiments. Authors can upload supporting documentation to indicate the use of appropriate reporting guidelines for health-related research (see EQUATOR Network), life science research (see the BioSharing Information Resource), or the ARRIVE guidelines for reporting work involving animal research. Where applicable, authors should refer to any relevant reporting standards documents in this form.

If you have any questions, please consult our Journal Policies and/or contact us: editorial@elifesciences.org.

Sample-size estimation

- You should state whether an appropriate sample size was computed when the study was being designed
- You should state the statistical method of sample size computation and any required assumptions
- If no explicit power analysis was used, you should describe how you decided what sample (replicate) size (number) to use

Please outline where this information can be found within the submission (e.g., sections or figure legends), or explain why this information doesn't apply to your submission:

All subjects in this study were from open-sourced datasets. We replicated the identifiability analysis across three datasets with more than 480 subjects in total and used more than 1000 subjects for analyzing the relationships between the brain asymmetry and sex, handedness, and cognition. For heritability analysis, we used 138 monozygotic (MZ) twin pairs, 79 dizygotic (DZ) twin pairs, and 160 of their non-twin siblings, which corresponds to the largest publicly available dataset of twins with MRI.

Replicates

- You should report how often each experiment was performed
- You should include a definition of biological versus technical replication
- The data obtained should be provided and sufficient information should be provided to indicate the number of independent biological and/or technical replicates
- If you encountered any outliers, you should describe how these were handled
- Criteria for exclusion/inclusion of data should be clearly stated
- High-throughput sequence data should be uploaded before submission, with a private link for reviewers provided (these are available from both GEO and ArrayExpress)

Please outline where this information can be found within the submission (e.g., sections or figure legends), or explain why this information doesn't apply to your submission:

We replicated the identifiability analysis across three datasets. We used the shortest inter-sessional interval in our main analysis and also replicated the analysis using the longest inter-sessional intervals in two of these datasets (another one only has one inter-sessional interval). When comparing the identifiability score of the functional connectivity, we used two atlases to replicate the functional connectivity.

Statistical reporting

- Statistical analysis methods should be described and justified
- Raw data should be presented in figures whenever informative to do so (typically when N per group is less than 10)
- For each experiment, you should identify the statistical tests used, exact values of N, definitions of center, methods of multiple test correction, and dispersion and precision measures (e.g., mean, median, SD, SEM, confidence intervals; and, for the major substantive results, a measure of effect size (e.g., Pearson's r, Cohen's d)
- Report exact p-values wherever possible alongside the summary statistics and 95% confidence intervals. These should be reported for all key questions and not only when the p-value is less than 0.05.

Please outline where this information can be found within the submission (e.g., sections or figure legends), or explain why this information doesn't apply to your submission:

> All analysis methods are described in the "Materials and methods" section, and the statistical inference procedures are described in the "Statistical Analysis" section.

(For large datasets, or papers with a very large number of statistical tests, you may upload a single table file with tests, Ns, etc., with reference to sections in the manuscript.)

Group allocation

- Indicate how samples were allocated into experimental groups (in the case of clinical studies, please specify allocation to treatment method); if randomization was used, please also state if restricted randomization was applied
- Indicate if masking was used during group allocation, data collection and/or data analysis

Please outline where this information can be found within the submission (e.g., sections or figure legends), or explain why this information doesn't apply to your submission:

> We used 233 subjects from the OASIS-3, 208 subjects from the ADNI, and 45 subjects from the HCP test-retest subsample for identifiability analysis. For analyzing the relationships between sex and brain asymmetry, we used data from 504 males and 602 females from the HCP dataset. For heritability analysis, we used 138 MZ twin pairs, 79 DZ twin pairs, and 160 of their non-twin siblings. Subject numbers of datasets and analyses are described in the section of each dataset of the "Materials and methods".

Additional data files ("source data")

- We encourage you to upload relevant additional data files, such as numerical data that are represented as a graph in a figure, or as a summary table
- Where provided, these should be in the most useful format, and they can be uploaded as "Source data" files linked to a main figure or table
- Include model definition files including the full list of parameters used
- Include code used for data analysis (e.g., R, MatLab)
- Avoid stating that data files are "available upon request"

Please indicate the figures or tables for which source data files have been provided:

eLife Sciences Publications, Ltd is a limited liability non-profit non-stock corporation incorporated in the State of Delaware, USA, with company number 5030732, and is registered in the UK with company number FC030576 and branch number BR015634 at the address Westbrook Centre, Milton Road, Cambridge CB4 1YG.

Model definition is described in the “Materials and methods” section. All code and dependent toolbox used in this study can be found at: https://github.com/cyctbdbw/Shape-Asymmetry-Signature. The code of shape-DNA can be found at: http://reuter.mit.edu/software/shapedna/. The OASIS-3 dataset is available under https://www.oasis-brains.org/. The ADNI dataset is available under https://adni.loni.usc.edu. The HCP dataset is available under https://db.humanconnectome.org/.

eLife Sciences Publications, Ltd is a limited liability non-profit non-stock corporation incorporated in the State of Delaware, USA, with company number 5030732, and is registered in the UK with company number FC030576 and branch number BR015634 at the address Westbrook Centre, Milton Road, Cambridge CB4 1YG.
